# Association between induction of the self-management system for preventing readmission and disease severity and length of readmission in patients with heart failure

**DOI:** 10.1186/s13104-021-05864-6

**Published:** 2021-12-18

**Authors:** Eisaku Nakane, Takao Kato, Nozomi Tanaka, Tomoari Kuriyama, Koki Kimura, Shushi Nishiwaki, Toka Hamaguchi, Yusuke Morita, Yuhei Yamaji, Yoshisumi Haruna, Tetsuya Haruna, Moriaki Inoko

**Affiliations:** 1 Cardiovascular Center, Tazuke Kofukai Medical Research Institute, Kitano Hospital, 2-4-20 Ohgimachi, Kita-ku, Osaka City, 530-8480 Japan; 2 Department of Cardiovascular Medicine, Graduate School of Medicine, Kyoto University, 54 Shogoin Kawahara-cho, Sakyo-ku, Kyoto, 606-8507 Japan

**Keywords:** Self-care management, Heart failure, Readmission, Rehospitalization

## Abstract

**Objective:**

We recently developed the self-management system using the HF points and instructions to visit hospitals or clinics when the points exceed the pre-specified levels. We found that the self-management system decreased the hospitalization for HF with an increase in unplanned visits and early intervention in the outpatient department. However, it is unclear whether we managed severe HF outpatients who should have been hospitalized. In this study, we aimed to compare HF severity in rehospitalized patients with regard to self-management system use.

**Results:**

We retrospectively enrolled 306 patients (153 patients each in the system user and non-user groups) using propensity scores (PS). We compared HF severity and length of readmission in rehospitalized patients in both groups. During the 1-year follow-up period, 24 system users and 43 non-system users in the PS-matched cohort were hospitalized. There were no significant differences between the groups in terms of brain natriuretic peptide levels at readmission, maximum daily intravenous furosemide dose, percentage of patients requiring intravenous inotropes, duration of hospital stay and in-hospital mortality. These results suggest that the HF severity in rehospitalized patients was not different between the two groups.

**Supplementary Information:**

The online version contains supplementary material available at 10.1186/s13104-021-05864-6.

## Introduction

Self-care maintenance and management are necessary to prevent rehospitalizations for heart failure (HF). It is important for patients and caregivers to manage worsening HF [1]. However, it is difficult for patients and caregivers to conclude whether HF is worsening, indicating the need to see a doctor [2, 3]. Therefore, we have developed a new system to make self-care management as easy as possible [4]. The new HF self-care system provided HF “points” for weight and clinical symptoms, and the total scores were correlated with appropriate consultation times for both patients and healthcare providers [4]. The HF points for each component are as follows: 1 point, if there is at least one presenting HF symptom (dyspnea on exertion, edema, cough, and appetite loss); 3 points, if the body weight exceeds the set weight limit; 4 points, if the heart rate is ≥ 120 beats per minute (bpm); and 5 points, if there is dyspnea at rest [4]. Patients with 3 HF points were instructed to visit the nearest outpatient clinic within 1 week, and those with 4 HF points were instructed to visit their physician on the same or the next day because of possible worsening HF [4]. Since hospitalization was highly indicated for patients with ≥ 5 points, they were instructed to visit the nearest emergency department [4]. Home and outpatient nursing staff can help patients in recognizing worsening HF and support them in receiving medical care [4]. We recently reported the benefits of this system using a propensity score (PS)-matched cohort [4]. The composite endpoint of all-cause mortality and HF hospitalization rate was significantly lower in system users after 1 year, primarily by reduction in the latter [4]. The usefulness of the new HF self-care system for preventing HF readmission was elucidated in the previous study [4]. The number of unscheduled outpatient visits and early interventions were higher in the user group [4]. However, it is unclear whether there were very severe HF outpatients who required hospitalization.

In this study, we aimed to compare HF severity and length of readmission between the same cohort of system and non-system users from our previous study. This is a post hoc sub-study of the previous study in which the cumulative incidence and risk for readmission were analyzed; however, the severity of HF was not assessed [4]. This study provided additional data on HF severity when the patients were re-hospitalized.

## Main text

### Methods

#### Study design

**Fig. 1:**
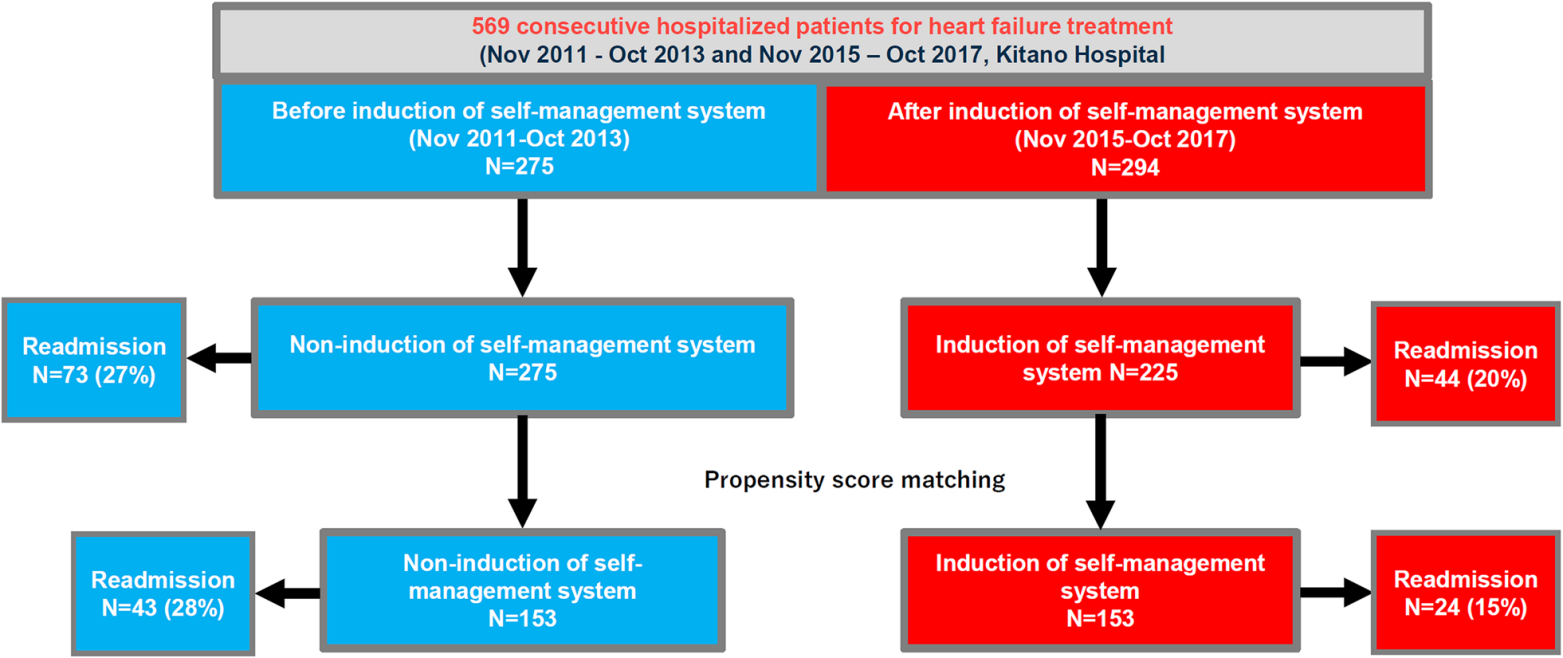
Patient flowchart

Among the 569 consecutive patients with HF admitted to Kitano Hospital, 275 and 294 patients were admitted between November 2011 and October 2013 (before system induction) and between November 2015 and October 2017 (after system induction), respectively, and were subsequently matched using PS [4]. Clinical follow-up data from all patients were collected in October 2017. Data analysis was conducted in August 2020. In the present analysis, we compared HF severity in readmitted self-management system users and non-users in the PS-matched cohort and entire cohort (Fig. 1).

### Ethics

The study protocol conformed to the ethical guidelines of the 1975 Declaration of Helsinki and was approved by the Institutional Review Board of Kitano Hospital (P190600100). The requirement of informed consent was waived because of the retrospective nature of the study. We disclosed all study details to the public using an opt-out method and clearly informed the patients of their right to refuse enrollment.

### Self-care management system

In the self-care HF assessment sheet described elsewhere [4], the patients’ weight and clinical symptoms are scored using “HF points”. The appropriate consultation times based on the scores were clarified to both patients and healthcare providers, described detailed previously [4]. We introduced the system to patients hospitalized for HF through a team conference at the beginning of their admission and to the patients’ cohabitants, nearby family members, caregivers, or nurses who can perform the assessment at least once a week for those who were unable to self-manage [4]. Early intervention was defined as escalation of oral and intravenous diuretics at the outpatient department or shortened outpatient visit intervals without any hospitalization [4].

### Primary outcome measures

The outcome measure in the current analysis was HF severity at readmission and length of readmission along with in-hospital mortality. HF severity in each group was evaluated using brain natriuretic peptide (BNP) levels at readmission, the maximum daily intravenous furosemide dose, and percentage of patients requiring intravenous inotrope. Data were collected retrospectively by chart review of each patient.

### Statistical analysis

We used a PS-matched cohort study design to balance the baseline characteristics between system users and non-users as previously reported [4]. In brief, a logistic regression model was developed to make the PS with 19 baseline variables clinically relevant to the induction of the self-management program to balance the baseline characteristics associated with users and non-users. We matched the patients based on the estimated PS using greedy-matching technique (Fig. 1 and Additional file 1: Method). We then evaluated the differences between rehospitalized patients in the two groups. Categorical variables, expressed as numbers with percentages, were compared using the chi-square test, and continuous variables, expressed as means with standard deviations or medians with 25–75th percentiles (interquartile range: IQR), were compared using Student’s t-test when normally distributed or the Wilcoxon rank-sum test when non-normally distributed. All statistical analyses were performed by physicians using JMP 14.0 (SAS Institute Inc., Cary, NC, USA) and SAS 9.4 (SAS Institute Inc., Cary, NC, USA). Statistical significance was set at P < 0.05.

### Results

#### Patient characteristics

During the 1-year follow-up of the PS-matched cohort, 43 (28%) system non-users and 24 (15%) users were admitted due to HF [4]. There were no significant differences in patient characteristics between the groups (Table 1). Unplanned visits and early interventions were more often in the user group than in the non-user group (Table 1). The trend of the patients from the entire cohort was almost consistent with that of the PS-matched cohort (Table 1).

**Table 1:** Study population characteristics derived from the propensity score-matched and entire cohorts

Variables	Propensity score-matched cohort			Entire cohort		
	Rehospitalized patients			Rehospitalized patients		
	User (N = 24)	Non-user (N = 43)	P value	User (N = 44)	Non-user (N = 73)	P value
Clinical characteristics						
Age, years	80.5 [76–84]	78.0 [69–84]	0.4874	80.5 [74–85]	78.0 [71–83]	0.216
Age > 80 years	13 (54%)	20 (47%)	0.615	23 (52%)	33 (45%)	0.567
Men	15 (63%)	21 (49%)	0.317	22 (50%)	33 (45%)	0.851
Etiology						
Ischemic heart disease	8 (33%)	19 (44%)	0.444	14 (32%)	32 (44%)	0.243
Valvular heart disease	11 (46%)	24 (56%)	0.457	21 (48%)	35 (48%)	1.000
Dilated cardiomyopathy	1 (4.2%)	5 (12%)	0.408	3 (6.8%)	8 (11%)	0.532
Medical history						
Atrial fibrillation or flutter	12 (50%)	24 (56%)	0.799	21 (48%)	38 (52%)	0.705
Cardiac resynchronization therapy	1 (4.2%)	3 (7.0%)	1.000	3 (6.8%)	4 (5.5%)	1.000
Implantable cardioverter defibrillator	0 (0%)	0 (0%)	1.000	2 (4.6%)	0 (0.0%)	0.139
Diabetes mellitus	9 (38%)	12 (28%)	0.426	14 (32%)	24 (33%)	1.000
Prior stroke	6 (25%)	13 (30%)	0.780	9 (20%)	18 (25%)	0.657
Chronic obstructive lung disease	6 (25%)	4 (9.3%)	0.149	10 (23%)	6 (8.2%)	0.049
Malignancy	7 (29%)	5 (12%)	0.099	10 (23%)	13 (18%)	0.632
Dementia	2 (8.3%)	3 (7.0%)	1.000	8 (18%)	4 (5.5%)	0.055
Vital signs at initial admission						
Systolic blood pressure, mmHg	121 ± 27.1	113 ± 18.7	0.347	118 ± 25.8	115 ± 20.7	0.782
Diastolic blood pressure, mmHg	65.9 ± 14.2	63.4 ± 13.6	0.431	65.8 ± 13,1	64.3 ± 14.1	0.451
Heart rate, bpm	70.4 ± 10.6	73.6 ± 12.1	0.364	71.3 ± 11.9	74.1 ± 12.2	0.242
Tests at initial admission						
Left ventricular ejection fraction (LVEF), %	44.0 ± 14.8	44.7 ± 17.8	0.759	41.9 ± 14.6	45.6 ± 16.3	0.204
LVEF < 40%	9 (38%)	22 (51%)	0.317	18 (41%)	32 (44%)	0.848
Estimated glomerular filtration rate (GFR), mL/min/1.73m^2^	34 [22–41]	30 [16–53]	0.901	33 [21–41]	26 [16–48]	0.648
Estimated GFR < 30 mL/min/1.73m^2^	11 (46%)	21 (49%)	1.000	20 (45%)	38 (52%)	0.568
Serum albumin < 3.5 g/dL	4 (17%)	6 (14%)	0.737	7 (16%)	13 (18%)	1.000
Hemoglobin, g/dL	12[10–13]	11[10–12]	0.374	11 [9.7–13]	11 [9.3–12]	0.095
Hemoglobin < 13 g/dL	17 (71%)	39 (91%)	0.046	33 (75%)	69 (94%)	0.004
Concomitant treatment						
β blockers	17 (71%)	27 (63%)	0.597	29 (66%)	41 (56%)	0.335
Angiotensin converting enzyme inhibitors or angiotensin receptor blockers	16 (67%)	35 (81%)	0.234	33 (75%)	52 (71%)	0.831
Aldosterone antagonists	12 (50%)	19 (44%)	0.799	24 (55%)	31 (42%)	0.252
Loop diuretics	21 (88%)	36 (84%)	1.000	40 (91%)	60 (82%)	0.280
Thiazides	4 (17%)	9 (21%)	0.757	8 (18%)	14 (19%)	1.000
Tolvaptan	4 (17%)	5 (12%)	0.711	19 (43%)	5 (6.9%)	< 0.001
Inotropic agents	4 (17%)	4 (9.3%)	0.443	5 (11%)	7 (9.6%)	0.762
Statins	10 (42%)	17 (40%)	1.000	18 (41%)	29 (40%)	1.000
Calcium antagonists	9 (38%)	14 (33%)	0.790	15 (34%)	31 (42%)	0.436
Multiple heart failure readmission (> 3times)	6 (25%)	5 (12%)	0.182	11 (25%)	9 (12%)	0.127
First heart failure admission	12 (50%)	26 (60%)	0.449	20 (45%)	41 (56%)	0.340
Living alone	5 (21%)	11 (26%)	0.770	13 (30%)	15 (21%)	0.275
Unplanned ambulatory visits after initial admission						
No visit	2 (8%)	5 (12%)	0.021	5 (11%)	10 (14%)	0.009
One time	15 (63%)	37 (86%)		25 (58%)	58 (80%)	
Twice	4 (17%)	1 (2%)		8 (18%)	4 (5%)	
Three times	2 (8%)	0 (0%)		5 (11%)	1 (1%)	
Four times or more	1 (4%)	0 (0%)		1 (2%)	0 (0%)	
Early intervention after initial admission^*^						
None	18 (74%)	42 (98%)	0.008	31 (71%)	69 (95%)	0.001
One time	3 (13%)	1 (2%)		9 (20%)	4 (5%)	
Twice	3 (13%)	0 (0%)		4 (9%)	0 (0%)	
Three times	0 (0%)	0 (0%)		0 (0%)	0 (0%)	

^*^Early intervention was defined as escalation of oral and intravenous diuretics at the outpatient department or shortened outpatient visit intervals without any hospitalization

### Outcome measures

The BNP levels at re-admission were not different between the groups (763 (IQR 516–1428) pg/ml in user group and 628 (IQR 435–1502) pg/ml in non-user group, P = 0.707). The maximum daily dose of intravenous furosemide and percentages of patients requiring intravenous inotropes were not different between the group (Table 2). The readmission length was 15.5 (IQR 12.3–26) days in user group and 15 (IQR 11–27) days in non-user group, respectively (P = 0.958). In-hospital mortality was 4.2% (1/24) and 9.3% (4/43) in the user and non-user groups, respectively (P = 0.647; Table 2).

**Table 2:** Outcome measures

Variables	Propensity score-matched cohort			Entire cohort		
	Rehospitalized patients			Rehospitalized patients		
	User (N = 24)	Non-user (N = 43)	P value	User (N = 44)	Non-user (N = 73)	P value
BNP value at rehospitalization (pg/ml)	763 (516–1428)	628 (435–1502)	0.707	984 (512–1493)	680 (376–1580)	0.403
The maximum daily dose of intravenous furosemide (mg)	15 (10–35)	10 (0–25)	0.275	20 (10–40)	10 (0–40)	0.192
Patients requiring intravenous inotropes, n (%)	13 (30%)	21 (29%)	1.000	13 (30%)	21 (29%)	1.000
The length of rehospitalization (day)	15.5 (12.3–26)	15 (11–27)	0.958	14.5 (12–25.3)	19 (13–34)	0.242
In-hospital mortality, n (%)	1 (4.2%)	4 (9.3%)	0.647	3 (6.8%)	7 (9.6%)	0.741

### Discussion

This study showed no significant difference in HF severity and length of readmission between self-care management system users and non-users. Using the system did not seem to cause that patients who were necessary to hospitalizations remain unhospitalized. Our results indicated that using a self-care management system may be clinically relevant as more frequent unplanned ambulatory visits and early interventions at outpatient clinics [4] prevented progression to very severe HF, in conjunction with the previous study [4].

Observational studies or sub-analyses of randomized trials have illustrated the impact of previous hospitalization on long-term mortality in patients with HF in Western [5–7] and Asian countries [8, 9]. The relationship with mortality and hospitalization was incremental [9]. The admission itself is related to adverse events [10], and functional decline was observed in patients hospitalized for HF [11]. Preventing rehospitalization is critical in the management of HF and maintenance of activities of daily living. The threshold for rehospitalization can be influenced by not only HF severity and presence of comorbidities but also differences in protocols among hospitals, physicians, or localities. After introducing the self-management system in our hospital [4], HF severity, length of readmission, and mortality rate did not differ between in system users and non-users. Overall, cumulative incidences of all-cause death in this PS-matched cohort were not different between the two groups, as reported previously [4]. Although the study was a retrospective study, with different timeframes between the users and non-users, the threshold for rehospitalization at outpatient visits was not influenced by the self-management system use. However, we did not evaluate the overall in-hospital and outpatient costs, [12] thus further studies are warranted to address this issue. In conclusion, there was no significant difference in HF severity and length of readmission between self-care management system users and non-users.

## Limitations

This study has several limitations. First, this study was retrospective study; thus, there were no pre-specified criteria for hospitalization nor the pre-specified treatment regimen at outpatient. Second, the continuation and quality of the self-management system were not assessed. Third, data on practices at unplanned visits were not analyzed. Finally, data from unplanned visits to other health centers were not collected. Fourth, additional factors beyond HF severity and clinical practice, such as inter-physician and patient thresholds for hospitalization and environmental factors of the patients, may have contributed to rehospitalization.

## Supplementary Information

**Additional file 1**. Method. Details of self-care management system and propensity score matching.

## Data Availability

The data analyzed during this study are not publicly available as the secondary use of the qualitative data is limited by ethical committee. Questions about access to deidentified data should be addressed to the corresponding author.

## Abbreviations

BNP: Brain natriuretic peptides
HF: Heart failure
IQR: Interquartile range
PS: Propensity scores
