# Supplementary material for: Association between induction of the self-management system for preventing readmission and disease severity and length of readmission in patients with heart failure
Source: BMC Res Notes. 2021 Dec 18;14:452. doi: 10.1186/s13104-021-05864-6 (PMC8684164; doi:10.1186/s13104-021-05864-6)
Supplement: Supplementary file 1 — Additional file 1. Method. Details of self-care management system and propensity score matching. [file 13104_2021_5864_MOESM1_ESM.docx]

**Additional file**

Association Between Induction of the Self-management System for Preventing Readmission and Disease Severity and Length of Readmission in Patients with Heart Failure

Authors: Eisaku Nakane, Takao Kato, Nozomi Tanaka, Tomoari Kuriyama, Koki Kimura, Shushi Nishiwaki, Toka Hamaguchi, Yusuke Morita, Yuhei Yamaji, Yoshisumi Haruna, , Tetsuya Haruna, Moriaki Inoko

**Methods: Page 2-4**

**Additional Methods**

**Details of self-care management system**

To perform self-care management more easily and uniformly in patients with heart failure (HF), we developed and implemented a new self-care system to facilitate early ambulatory visits by clarifying the appropriate timing of patient visits in November 2015 for all patients admitted for HF treatment^1^. The HF point self-care assessment sheet is presented in Supplementary Figure 1A and 1B). The main feature of this sheet is that the weight and HF symptoms are scored using points, which we named “HF points”, and the timing of consultation is clarified to both patients and health-care providers. HF points are the summation of a specific component: 1 point, if there is each of the initial symptoms of HF (dyspnea on exertion, edema, cough, and appetite loss); 3 points, when the weight increases and exceeds the set body weight; 4 points, when the heart rate exceeds or is equal to 120 bpm (beats per minute); and 5 points in case of dyspnea at rest. We set the heart rate as high as 120 bpm to 4 points so that the occurrence of atrial fibrillation could be detected and treated at an early stage. The instructions for hospital or clinic visit according to the summed points are presented in Supplementary Figure 1C. We instructed the patients to visit the nearest physician outpatient clinic within 1 week if they had 3 HF points. If there was no deterioration of HF, the ideal body weight was re-evaluated and reset by physicians. However, if early HF symptoms (+1 point) were added to weight gain (3 points) (total of 4 points), the patient were instructed to visit the physician on the same or next day because HF is possible to be worsening. Since hospitalization was highly likely to be needed for patients with 5 points or more, patients with ≥5 points were instructed to visit a hospital’s emergency department. To prevent rehospitalization, the patients were strongly instructed to consult an outpatient clinic in case of 3 or 4 points in the mild symptomatic state (Supplementary Figure 1C). When we introduced the HF points to hospitalized patients, we decided whether to use this system through team conference early in the patient’s hospital admission. If a given patient could not use this system, we introduced this system to the patient’s cohabitants, their young family members living nearby, or caregivers or visiting nurses who could check the HF points by visiting the patient's home at least once a week. See more details in Nakane E et al. J Cardiol. 2021 Jan;77(1):48–56.^1^

**Propensity matching**

A logistic regression model was developed to make the PS for the induction of the self-management program with 19 baseline variables (age, number of prior hospitalizations, living alone, atrial fibrillation or flutter, implantable cardioverter defibrillator, dementia, left ventricular ejection fraction <40%, brain natriuretic peptides> 235 pg/ml as a median value, estimated glomerular filtration rate < 30 ml/min/1.73 m2, serum potassium >4.3 mEq/L as a median value, serum albumin < 3.5 g/dL, homoglobin < 13g/dL, and the use of beta-blocker use, angiotensin converting enzyme inhibitor or angiotensin 2 receptor blocker, loop diuretics, tolvaptan, inotropic agent, and calcium antagonist). The c-statistics were 0.748. See more details in Nakane E et al. J Cardiol. 2021 Jan;77(1):48–56.^1^

**References of the additional file**

1. Nakane E, Kato T, Tanaka N, Kuriyama T, Kimura K, Nishiwaki S, Hamaguchi T, Morita Y, Yamaji Y, Haruna Y, Haruna T, Inoko M. Association of the induction of a self-care management system with 1-year outcomes in patients hospitalized for heart failure. J Cardiol. 2021 Jan;**77**(1):48-56.
